# Highly Pathogenic Avian Influenza A Virus in Wild Migratory Birds, Qinghai Lake, China, 2022

**DOI:** 10.3201/eid3010.240460

**Published:** 2024-10

**Authors:** Xiaoqing Zhang, Jiaying Wu, Yanhai Wang, Mengchan Hao, Haizhou Liu, Sanling Fan, Juan Li, Jianqing Sun, Yubang He, Yuan Zhang, Jianjun Chen

**Affiliations:** Wuhan Institute of Virology, Wuhan, China (X. Zhang, J. Wu, Y. Wang, M. Hao, H. Liu, S. Fan, Y. Zhang, J. Chen);; University of Chinese Academy Sciences, Beijing, China (X. Zhang, J. Wu, Y. Wang, M. Hao, S. Fan);; Shandong First Medical University & Shandong Academy of Medical Sciences, Taian, China (J. Li);; Qinghai Lake National Nature Reserve, Qinghai, China (J. Sun, Y. He)

**Keywords:** Avian Influenza A, influenza, viruses, birds, H5N1, Clade 2.3.4.4b, Qinghai Lake, China

## Abstract

In July 2022, an outbreak of highly pathogenic avian influenza A(H5N1) virus clade 2.3.4.4b occurred among migratory birds at Qinghai Lake in China. The virus circulated in June, and reassortants emerged after its introduction into the area. Surveillance in 2023 showed that the virus did not establish a stable presence in wild waterfowl.

Qinghai Lake in China, situated at the intersection of the Central Asian and East Asian–Australasian Flyways, is the largest lake in the Qinghai–Tibet Plateau (*1*). This breeding and stopover area for migratory birds supports >200,000 waterfowl each year (*2*). Historically, 4 outbreaks of highly pathogenic avian influenza viruses (HPAIVs) at Qinghai Lake occurred in 2005 (*3*), 2009 (*4*), 2015 (*5*), and 2016 (*6*) during the breeding season (May–August). Since 2015, we have performed long-term avian influenza surveillance at Qinghai Lake during the breeding season. Our previous studies have reported HPAIV outbreaks of H5N1 clade 2.3.2.1c in 2015 and H5N8 clade 2.3.4.4b in 2016 at Qinghai Lake (*5*–*7*). Since late 2020, H5N1 clade 2.3.4.4b viruses, which are descendant of H5N8 clade 2.3.4.4b viruses, have emerged and become the dominant HPAIVs and caused outbreaks worldwide (*8*). We describe data collected from our ongoing surveillance of avian influenza in the Qinghai Lake area and record the introduction of clade H5N1 2.3.4.4b to Qinghai Lake birds in 2022.

## The Study

**Figure 1:**
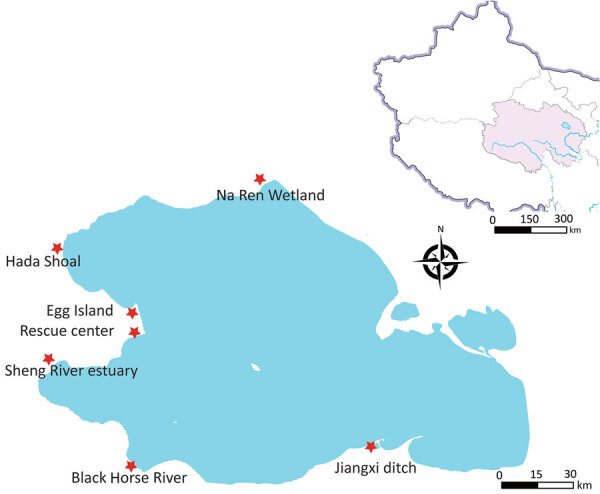
Fecal sample collection for the surveillance of avian influenza viruses at Qinghai Lake, China. Sampling sites in Qinghai Lake during the breeding season are shown. Inset map shows location of Qinghai Lake (blue) and the surrounding area in China.

**Figure 2:**
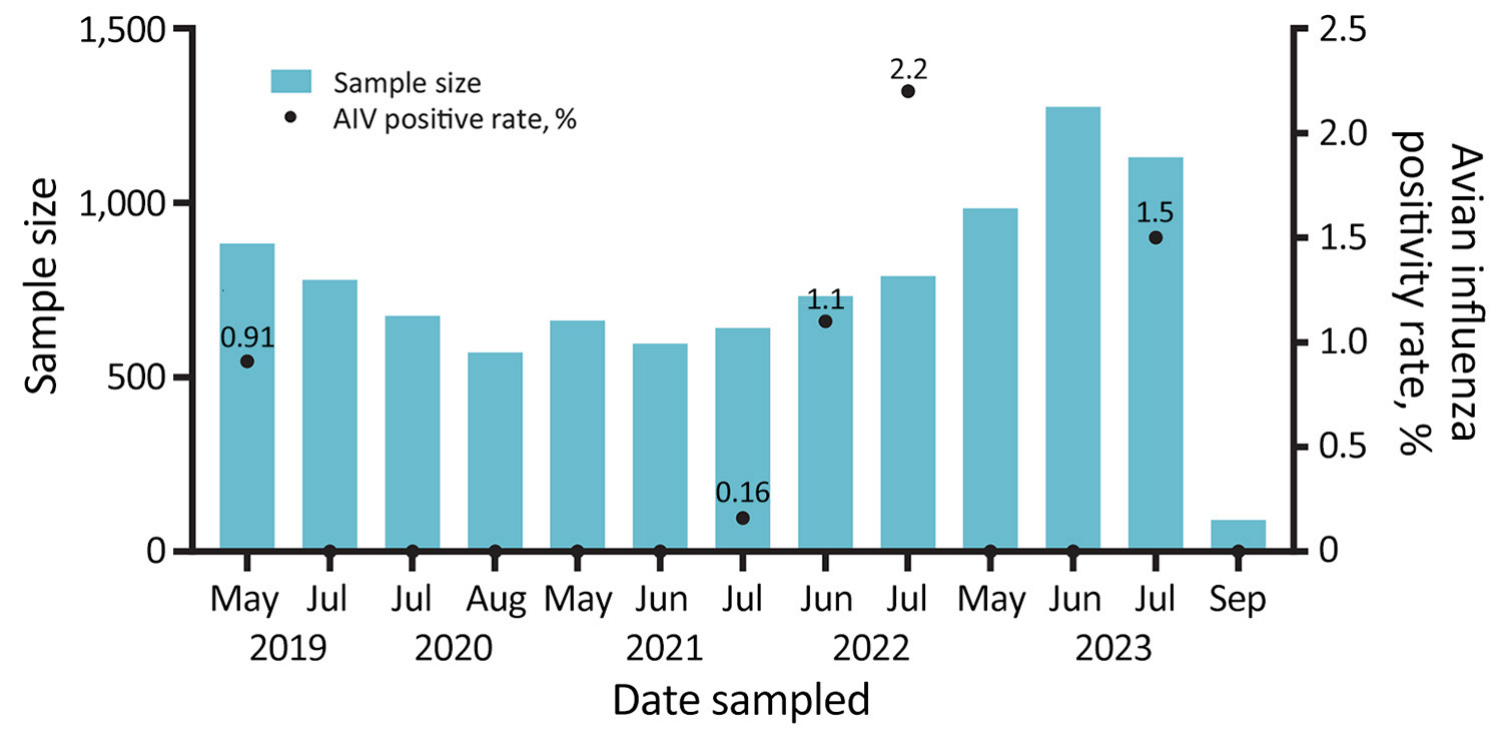
Monthly sample sizes and positivity rates from fecal sample collection for surveillance of AIVs at Qinghai Lake, China. AIV, avian influenza virus.

During 2019–2021, we collected fresh fecal samples annually from the wetlands around Qinghai Lake during the avian breeding season (Figure 1). No HPAIV was detected in 2019–2021, and only 8 strains of low pathogenic avian influenza viruses (LPAIVs) were isolated (Appendix Table 1). In June 2022, a highly pathogenic H5N1 virus emerged, and 8 strains were isolated from 726 fresh fecal samples (1.1%) (Figure 2). In addition, 5 decomposed bird carcasses were found at the sampling sites in June 2022, and H5N1 virus was isolated from a swab sample of a bar-headed goose (*Anser indicus*) carcass. In July 2022, an outbreak occurred, and >200 birds died. Our surveillance data showed the positivity rate of H5N1 virus in fecal samples was 0.68% (5/730) in July 2022 (Figure 2). H5N1 viruses were also isolated from tissue samples of the carcasses of 12 birds (Appendix Table 2). 

In 2023, we collected 3,481 fecal samples during May–September and obtained tissue samples from 9 wild bird carcasses (Appendix Table 2). No highly pathogenic H5 viruses were isolated from fecal or tissue samples in 2023, although several strains of low pathogenicity avian influenza viruses (LPAIVs) were isolated (Appendix Table 1). By using next-generation sequencing, we performed whole-genome sequencing of 25 H5N1 and other subtypes of avian influenza viruses isolated during 2022 and 2023 (Appendix).

To understand the genetic relationship between the Qinghai Lake H5N1 viruses and other viruses, we performed phylogenetic analysis of the 25 H5N1 strains and relevant sequences from public databases. The phylogenetic analysis of hemagglutinin (HA) showed that Qinghai Lake H5N1 belonged to clade 2.3.4.4b (Appendix Figure 1). Moreover, examination of the phylogenetic evolution trees of neuraminidase, polymerase basic 1 and 2, polymerase acidic (PA), nucleoprotein, matrix, and nonstructural protein genes indicated that Qinghai Lake H5N1 viruses clustered together with H5N1 strains isolated from wild birds and poultry in China (*9*), Japan (*10*), South Korea (*11*), Bangladesh (*12*), and Malaysia from the end of 2021 through the first half of 2023 (Appendix Figure 2). Those findings suggest a close genetic relationship between Qinghai Lake H5N1 viruses and strains from countries or regions along the East Asian­–Australasian and Central Asian migration flyways. 

**Figure 3:**
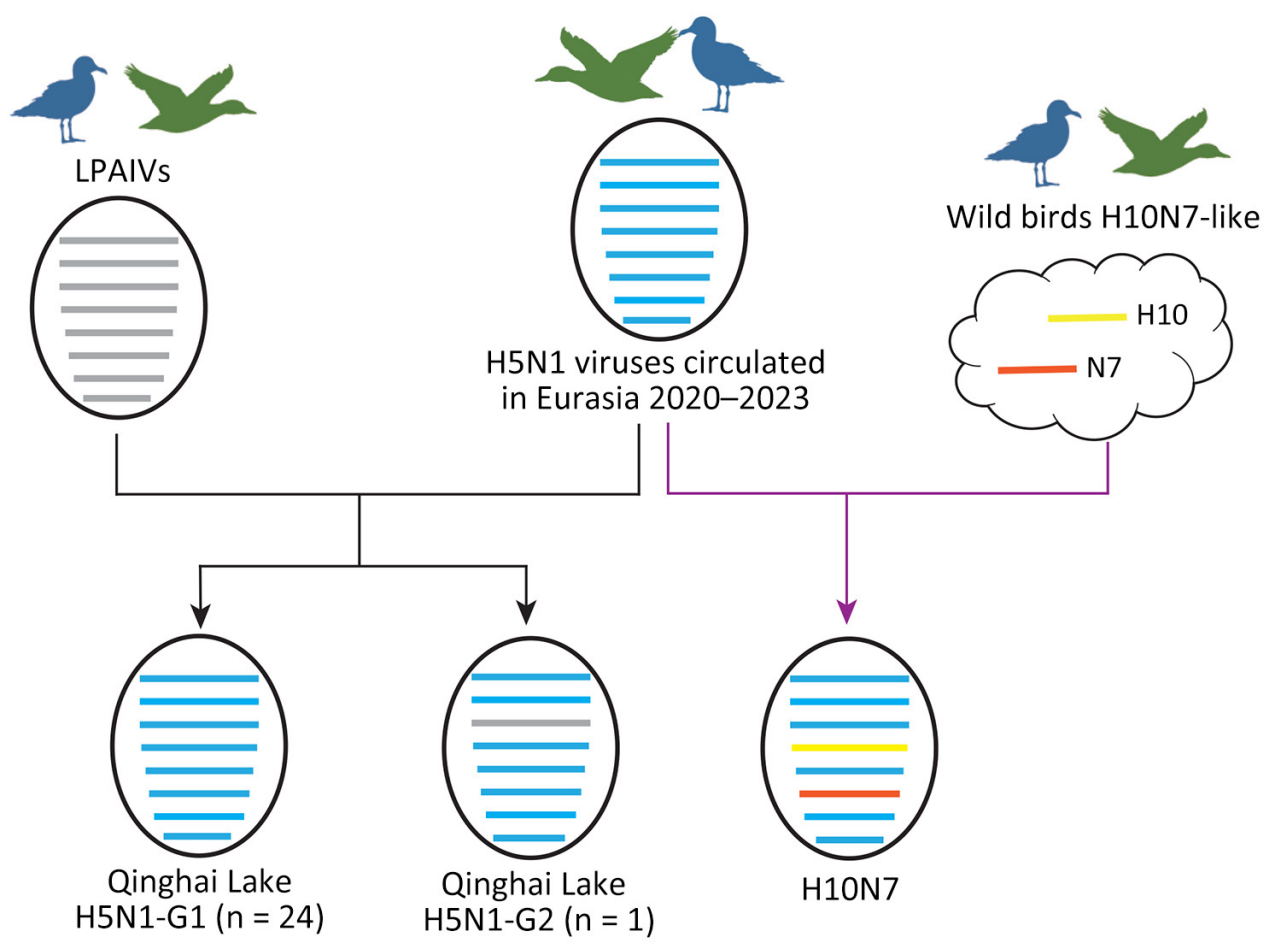
Hypothetical reassortment pathway of avian influenza virus H5N1 and H10N7 isolates collected at Qinghai Lake, China, in 2022. Virus particles are shown as ovals containing horizontal bars representing 8 gene segments (top to bottom: polymerase basic 1 and 2, polymerase acidic, hemagglutinin, nucleoprotein, neuraminidase, matrix, and nonstructural). The colors represent the genetic origin of reassortments found. G1, genotype 1; G2, genotype 2; LPAIVs, low pathogenic avian influenza viruses.

In the phylogenetic tree of the PA gene (Appendix Figure 2), the Qinghai Lake strains fell into 2 branches and only 1 strain (A/Bar-headed goose/Qinghai/06-225-2/2022 [H5N1]) clustered together with the LPAIV H10 strain isolated from Qinghai Lake in the same month, forming a separate monocluster with LPAIVs from China, Japan, South Korea, and Bangladesh. Analysis on the basis of the different sources of the PA gene revealed 2 genotypes among the 25 H5N1 strains (Figure 3); most H5N1 strains belonged to genotype G1 (n = 24) and only A/Bar-headed goose/Qinghai/06-225-2/2022 (H5N1) strains belonging to genotype G2 (n = 1). In the phylogenetic trees of 6 internal protein genes, H10N7 strain A/Bar-headed goose/Qinghai/06-JXG-1/2022, isolated from Qinghai Lake in June 2022, clustered together with the Qinghai Lake H5N1 strains (Appendix Figure 2), indicating reassortment between H5N1 and H10N7 viruses. The phylogenetic results showed that, after H5N1 virus was introduced into Qinghai Lake, reassortment occurred with LPAIVs in local wild birds. Of note, H5N1 acquired the PA gene from the low pathogenicity strain, leading to the emergence of a new genotype strain. The H10N7 strain obtained all 6 internal genes from H5N1 through reassortment (Figure 3).

Amino acid sequence analysis showed that all the 25 H5N1 viruses isolated in June and July 2022 were HPAIVs (Appendix Table 3). Those viruses contain multiple basic amino acids (REKRRKR/G) at the HA protein cleavage site. The HA proteins of those viruses had T160A mutations that were associated with enhanced binding ability to the α-2,6 receptor (*13*). In addition, amino acid mutations associated with increased virulence and replication in mammals have been identified in multiple proteins (Appendix Table 3).

**Figure 4:**
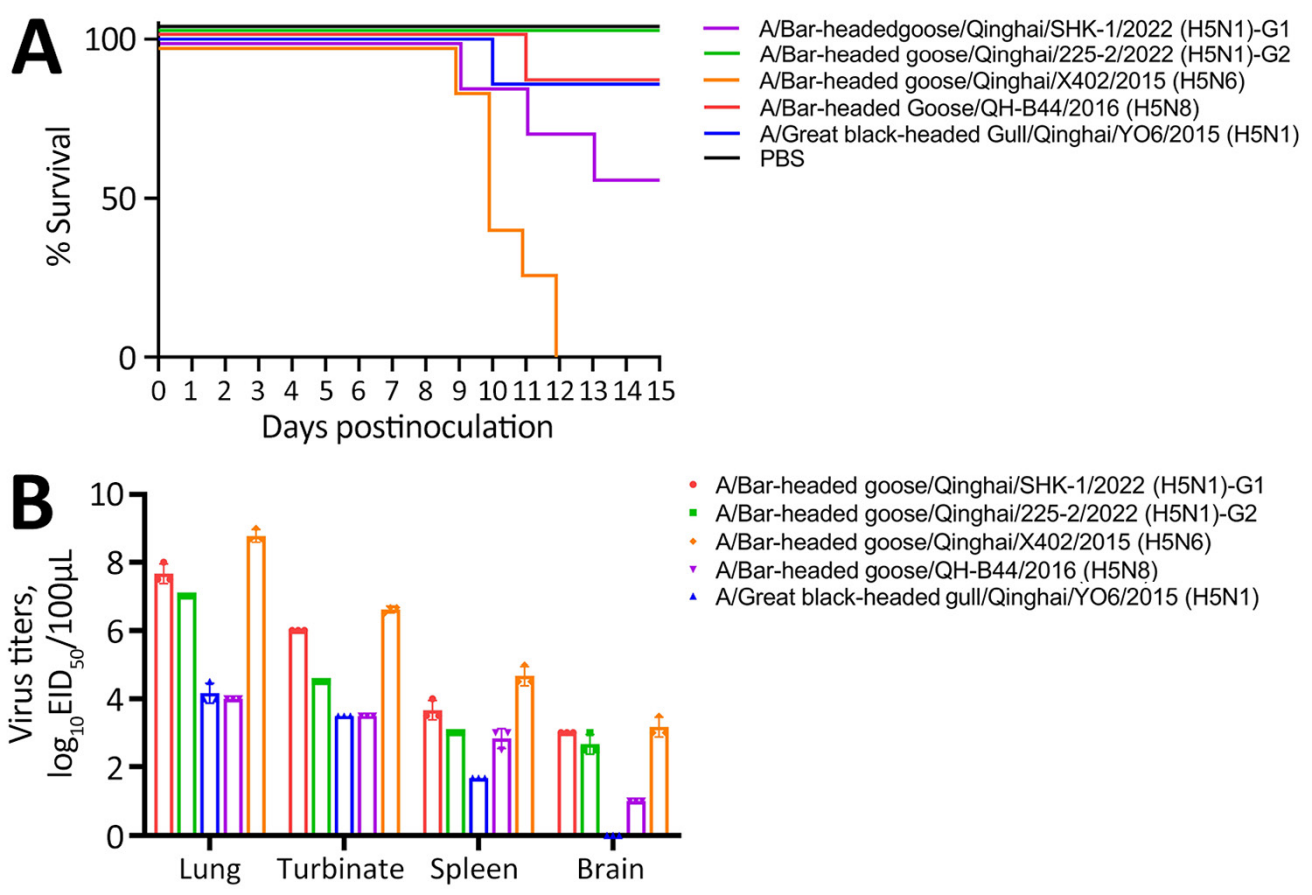
Mouse models of replication and pathogenicity of highly pathogenic H5 avian influenza viruses isolated from wild birds at Qinghai Lake, China. Each group of mice was inoculated intranasally at a dose of 10^6^ EID_50_ of H5N1 (3 strains), H5N8, and H5N6 viruses. Mice in the control group were inoculated with PBS. A) Kaplan–Meier survival curve. B) Organ viral titers determined at 3 days postinoculation by measuring EID_50_ in organ tissue from infected mice. Three mice from each group were euthanized for organ tissue collection. EID_50_, 50% egg infectious dose; PBS, phosphate-buffered saline.

We evaluated the pathogenicity of Qinghai Lake isolates in BALB/c mice. The isolates included were 2022 H5N1 (clade 2.3.4.4b), 2015 H5N1 (clade 2.3.2.1c), 2015 H5N6 (clade 2.3.4.4), and 2016 H5N8 (clade 2.3.4.4) strains isolated by our group during surveillance and previous outbreaks. Inoculation with the Qinghai Lake genotype G1 H5N1 strain from 2022 resulted in a ≈43% mortality rate (3/7) in mice, whereas all mice in the genotype G2 infection group survived (Figure 4, panel A), indicating differences in the pathogenicity of G1 and G2 genotype strains. After inoculation with the 2016 H5N8 and 2015 H5N1 strains, 1/7 mice died, whereas the 2015 H5N6 strain caused death in all inoculated mice (Figure 4, panel A). We performed a virus titer analysis of multiple organs and found the Qinghai Lake viruses could replicate in the lungs, nasal turbinates, and spleens of mice. The viruses could also replicate in mouse brains, except for the 2015 H5N1 (clade 2.3.2.1c) strain (Figure 4, panel B).

## Conclusions

Our surveillance data showed H5N1 clade 2.3.4.4b virus emerged in summer 2022 in the Qinghai Lake area. Because there is no poultry in the vicinity of Qinghai Lake, the virus was likely spread because of migratory birds, similar to the case for H5N8 in 2016 (*6*). In 2023, the H5N1 clade 2.3.4.4b virus was not detected, suggesting that this virus does not exhibit sustained circulation among wild birds at Qinghai Lake. Because the H5N1 clade 2.3.4.4b virus continues to circulate in other regions (*14*), it is possible for reintroduction to Qinghai Lake to cause an outbreak. Therefore, continuous surveillance of avian influenza virus in wild birds at Qinghai Lake is necessary.

The H5N1 clade 2.3.4.4b viruses isolated in this study had amino acid mutations associated with increased virulence and replication in mammals. Our animal experiment also demonstrated that genotype G1 of the H5N1 strain resulted in death in mice, suggesting that the virus has the potential to spill over to nonhuman mammals. Many livestock (mainly sheep, goats, and yaks) graze around Qinghai Lake, and wild birds and livestock often graze on the same grassland. A risk for transmission of H5N1 clade 2.3.4.4b virus from infected birds to livestock at Qinghai Lake exists, similar to bird-to-cow transmission of H5N1 clade 2.3.4.4b previously reported in the United States (*15*). Avian influenza virus surveillance should include livestock around Qinghai Lake.

Appendix Additional information for study of pathogenic avian influenza A virus in wild migratory birds, Qinghai Lake, China, 2022.

## References

[1] Prosser DJ, Cui P, Takekawa JY, Tang M, Hou Y, Collins BM, et al. Wild bird migration across the Qinghai-Tibetan plateau: a transmission route for highly pathogenic H5N1. PLoS One. 2011;6:e17622. 10.1371/journal.pone.0017622 21408010 PMC3052365

[2] Reserve QLNN. Qinghai Lake home to 200,000 migratory birds. 2018 [cited 2023 Aug 20] http://www.ecns.cn/hd/2018-08-20/detail-ifyxccrz0968790.shtml

[3] Liu J, Xiao H, Lei F, Zhu Q, Qin K, Zhang XW, et al. Highly pathogenic H5N1 influenza virus infection in migratory birds. Science. 2005;309:1206. 10.1126/science.1115273 16000410

[4] Hu X, Liu D, Wang M, Yang L, Wang M, Zhu Q, et al. Clade 2.3.2 avian influenza virus (H5N1), Qinghai Lake region, China, 2009-2010. Emerg Infect Dis. 2011;17:560–2. 10.3201/eid1703.100948 21392463 PMC3166005

[5] Bi Y, Chen J, Zhang Z, Li M, Cai T, Sharshov K, et al. Highly pathogenic avian influenza H5N1 Clade 2.3.2.1c virus in migratory birds, 2014-2015. Virol Sin. 2016;31:300–5. 10.1007/s12250-016-3750-4 27405930 PMC8193437

[6] Li M, Liu H, Bi Y, Sun J, Wong G, Liu D, et al. Highly pathogenic avian influenza A(H5N8) virus in wild migratory birds, Qinghai Lake, China. Emerg Infect Dis. 2017;23:637–41. 10.3201/eid2304.161866 28169827 PMC5367427

[7] Chen J, Liang B, Hu J, Liu H, Sun J, Li M, et al. Circulation, evolution and transmission of H5N8 virus, 2016-2018. J Infect. 2019;79:363–72. 10.1016/j.jinf.2019.07.005 31306679

[8] Xie R, Edwards KM, Wille M, Wei X, Wong SS, Zanin M, et al. The episodic resurgence of highly pathogenic avian influenza H5 virus. Nature. 2023;622:810–7. 10.1038/s41586-023-06631-2 37853121

[9] Cui P, Shi J, Wang C, Zhang Y, Xing X, Kong H, et al. Global dissemination of H5N1 influenza viruses bearing the clade 2.3.4.4b HA gene and biologic analysis of the ones detected in China. Emerg Microbes Infect. 2022;11:1693–704. 10.1080/22221751.2022.2088407 35699072 PMC9246030

[10] Soda K, Mekata H, Usui T, Ito H, Matsui Y, Yamada K, et al. Genetic and antigenic analyses of H5N8 and H5N1 subtypes high pathogenicity avian influenza viruses isolated from wild birds and poultry farms in Japan in the winter of 2021-2022. J Vet Med Sci. 2023;85:1180–9. 10.1292/jvms.23-0121 37766550 PMC10686771

[11] Cha RM, Lee YN, Park MJ, Baek YG, Shin JI, Jung CH, et al. Genetic characterization and pathogenesis of H5N1 high pathogenicity avian influenza virus isolated in South Korea during 2021–2022. Viruses. 2023;15:1403. 10.3390/v15061403 37376703 PMC10304347

[12] Barman S, Turner JCM, Kamrul Hasan M, Akhtar S, Jeevan T, Franks J, et al. Emergence of a new genotype of clade 2.3.4.4b H5N1 highly pathogenic avian influenza A viruses in Bangladesh. Emerg Microbes Infect. 2023;12:e2252510. 10.1080/22221751.2023.2252510 37622753 PMC10563617

[13] Linster M, van Boheemen S, de Graaf M, Schrauwen EJA, Lexmond P, Mänz B, et al. Identification, characterization, and natural selection of mutations driving airborne transmission of A/H5N1 virus. Cell. 2014;157:329–39. 10.1016/j.cell.2014.02.040 24725402 PMC4003409

[14] Adlhoch C, Fusaro A, Gonzales JL, Kuiken T, Mirinavičiūtė G, Niqueux É, et al.; European Food Safety Authority; European Centre for Disease Prevention and Control; European Union Reference Laboratory for Avian Influenza. Avian influenza overview September-December 2023. EFSA J. 2023;21:e8539. 38116102 10.2903/j.efsa.2023.8539 PMC10730024

[15] Oguzie JU, Marushchak LV, Shittu I, Lednicky JA, Miller AL, Hao H, et al. Avian influenza A(H5N1) virus among dairy cattle, Texas, USA. Emerg Infect Dis. 2024;30:1425–9. 10.3201/eid3007.240717 38848249 PMC11210641

