# Supplementary material for: Highly Pathogenic Avian Influenza A Virus in Wild Migratory Birds, Qinghai Lake, China, 2022
Source: Emerg Infect Dis. 2024 Oct;30(10):2135–9. doi: 10.3201/eid3010.240460 (PMC11431930; doi:10.3201/eid3010.240460)
Supplement: Appendix — Additional information for study of pathogenic avian influenza A virus in wild migratory birds, Qinghai Lake, China, 2022. [file 24-0460-Techapp-s1.pdf]

Article DOI: https://doi.org/10.3201/eid3010.240460

EID cannot ensure accessibility for supplementary materials supplied by authors. Readers who have difficulty accessing supplementary content should contact the authors for assistance.

Highly Pathogenic Avian Influenza A Virus in Wild Migratory Birds, Qinghai Lake, China, 2022

Appendix

Methods

Sample collection

Feces from healthy birds, cloacal swabs, and carcass organs were collected for viral isolation. Fresh and well-separated droppings were sampled, and fecal swabs were obtained using sterile swabs. Each sample was placed in a vial containing 2 mL of viral transport medium, stored at 2–8°C, and shipped to the laboratory within 12 h for further analysis. The tissue samples were collected from the laboratory.

Virus isolation and sequencing

Before inoculation, swabs were thoroughly mixed with the viral transport medium, and centrifuged at 8000 rpm and 4°C for 10 min until further use. Tissues and organs were homogenized in 1 mL cold PBS under sterile conditions. The solid debris in the swab and tissue samples were then pelleted by centrifugation at 8000 rpm and 4°C for 10 min. The precipitate was discarded, and antimicrobial agents were added to the processed supernatant, resulting in a final concentration of 100U/ml for penicillin, 0.1mg/ml for streptomycin, and 0.25 μ g/ml for amphotericin B.

Treated samples were inoculated into the allantoic cavities of 10-day-old specific pathogen-free embryonated eggs. After incubation at 37°C for 48–72 h, the allantoic fluid of inoculated eggs was collected and tested for the presence of HA-titer using 1% chicken red blood cells. HA-titer-positive samples were then confirmed for AIVs through reverse transcription PCR, as previously described (*1*). Using next-generation sequencing combined with Sanger sequencing, we performed whole-genome sequencing of 25 H5N1, 1 H2N3, 2 H3N8, 3 H4N6, 2 H12N5, 5 H10N5, 5 H10N9, 6 H10N7, 1H10N8 and 3 H10N1 strains, isolated between 2022 and 2023, as previously described (*2*). Whole-genome sequences of 25 strains of Qinghai Lake H5N1 influenza and other subtype LPAIVs isolates were determined and deposited in the Global Initiative on Sharing All Influenza Data (GISAID) (accession nos. EPI19004930, EPI19004931, EPI19004932, EPI19004933, EPI19004934, EPI19004935, EPI19004936, EPI19004937, EPI19004938, EPI19004939, EPI19004940, EPI19004941, EPI19004942, EPI19004943, EPI19004944, EPI19004945, EPI19004946, EPI19004947, EPI19004948, EPI19004949, EPI19004950, EPI19004951, EPI19004952,EPI19004953, EPI19004954, EPI19004955, EPI19004956, EPI19004957, EPI19004958, EPI19004959, EPI19004960, EPI19004961, EPI19004962, EPI19004963, EPI19214809, EPI19214810, EPI19214811, EPI19214812, EPI19214813, EPI19214814, EPI19214815, EPI19214816, EPI19214817, EPI19214818, EPI19214961, EPI19214969, EPI19214962, EPI19214963, EPI19214964, EPI19214965, EPI19214966, EPI19214967, EPI19214968).

Genetic analysis

All sequence data of the influenza A virus available from the GISAID and GenBank databases were obtained and combined into one database on August 18, 2023. Analysis datasets were generated by querying each nucleotide sequence of the QH-H5N1 virus against a combined database using the BLASTN program with default parameters. Sequences of the top 1000 hits were collected, including all QH-H5N1-related viruses and the closest virus. Identical sequences and sequences without a clear subtype or collection date were excluded from each segmented

dataset. These formed new subsets of data used for further phylogenetic analyses. Alignment of each gene fragment in the dataset was achieved using the Mafft software (v7.508), and redundant non-CDS base pairs were deleted after alignment.

The best model for tree construction was selected using IQTREE (v1.6.12)'s MF (Model Finder) model, and the bootstrap value was set to 1000 for the final ML tree drawing using the best model. Optimal nucleotide substitution model for each gene was as follows: PB2, GTR+F+I+G4; PB1, GTR+F+I+G4; PA, GTR+F+R3; HA, GTR+F+G4; NP, TVM+F+I+G4; N1, TVM+F+I+G4; M, GTR+F+I+R3; NS, GTR+F+G4.

Animal experiments

Experiments using live HP H5 viruses were conducted in a biosafety level 3 facility at the Wuhan Institute of Virology (WIV), CAS. All animal experiment procedures were approved in advance by the ethics committees of Wuhan Institute of Virology, Chinese Academy of Sciences.

During our routine surveillance of AIV in Qinghai Lake, different clades of H5 strains, including 2.3.4.4b H5N1, 2.3.2.1c H5N1, 2.3.4.4a H5N6, and 2.3.4.4b H5N8 were isolated. In this study, several representative stains were selected for subsequent animal pathogenetic experiment. These strains were A/Bar-headed goose/Qinghai/SHK-1/2022 (H5N1) (clade 2.3.4.4b), A/Bar-headed goose/Qinghai/225–2/2022 (H5N1) (clade 2.3.4.4b), A/Great black-headed Gull/Qinghai/YO6/2015 (H5N1) (clade 2.3.2.1c), A/Bar-headed goose/Qinghai/X402/2015 (H5N6) (clade 2.3.4.4), and A/Bar-headed Goose/QH-B44/2016 (H5N8) (clade 2.3.4.4b). Animal experiments were performed as previously described (*3*). Briefly, five strains of the virus were diluted to 10^6 EID_{50}/20 μL, and ten 6-week-old SPF BALB/c mice were intranasally infected with 20 μL of each viral strains under anesthesia. On the third day after infection, three mice from each group were randomly euthanized, and the brain, spleen, lung, and nasal turbinate bones were collected for virus titration. The remaining

seven mice were weighed daily until day 14 and euthanized. The virus titer in each organ was determined using chicken embryo eggs, and the replication titer of the organs was calculated according to the Reed–Muench method (*4*).

References

1. Chen J, Liang B, Hu J, Liu H, Sun J, Li M, et al. Circulation, evolution and transmission of H5N8 virus, 2016-2018. J Infect. 2019;79:363–72. PubMed https://doi.org/10.1016/j.jinf.2019.07.005

2. Bi Y, Chen Q, Wang Q, Chen J, Jin T, Wong G, et al. Genesis, evolution and prevalence of H5N6 avian influenza viruses in China. Cell Host Microbe. 2016;20:810–21. PubMed https://doi.org/10.1016/j.chom.2016.10.022

3. Liu H, Xiong C, Chen J, Chen G, Zhang J, Li Y, et al. Two genetically diverse H7N7 avian influenza viruses isolated from migratory birds in central China. Emerg Microbes Infect. 2018;7:62. PubMed https://doi.org/10.1038/s41426-018-0064-7

4. Lei C, Yang J, Hu J, Sun X. On the calculation of $TCID_{50}$ for quantitation of virus infectivity. Virol Sin. 2021;36:141–4. PubMed https://doi.org/10.1007/s12250-020-00230-5

5. Schrauwen EJA, Herfst S, Leijten LM, van Run P, Bestebroer TM, Linster M, et al. The multibasic cleavage site in H5N1 virus is critical for systemic spread along the olfactory and hematogenous routes in ferrets. J Virol. 2012;86:3975–84. PubMed https://doi.org/10.1128/JVI.06828-11

6. Yamada S, Suzuki Y, Suzuki T, Le MQ, Nidom CA, Sakai-Tagawa Y, et al. Haemagglutinin mutations responsible for the binding of H5N1 influenza A viruses to human-type receptors. Nature. 2006;444:378–82. PubMed https://doi.org/10.1038/nature05264

7. Linster M, van Boheemen S, de Graaf M, Schrauwen EJA, Lexmond P, Mänz B, et al. Identification, characterization, and natural selection of mutations driving airborne transmission of A/H5N1 virus. Cell. 2014;157:329–39. PubMed https://doi.org/10.1016/j.cell.2014.02.040

8. Pabbaraju K, Tellier R, Wong S, Li Y, Bastien N, Tang JW, et al. Full-genome analysis of avian influenza A(H5N1) virus from a human, North America, 2013. Emerg Infect Dis. 2014;20:887–91 PubMed https://doi.org/10.3201/eid2005.140164

9. Arai Y, Kawashita N, Ibrahim MS, Elgendy EM, Daidoji T, Ono T, et al. PB2 mutations arising during H9N2 influenza evolution in the Middle East confer enhanced replication and growth in mammals. PLoS Pathog. 2019;15:e1007919. PubMed https://doi.org/10.1371/journal.ppat.1007919

10. Elgendy EM, Arai Y, Kawashita N, Daidoji T, Takagi T, Ibrahim MS, et al. Identification of polymerase gene mutations that affect viral replication in H5N1 influenza viruses isolated from pigeons. J Gen Virol. 2017;98:6–17. PubMed https://doi.org/10.1099/jgv.0.000674

11. Feng X, Wang Z, Shi J, Deng G, Kong H, Tao S, et al. Glycine at position 622 in PB1 contributes to the virulence of H5N1 avian influenza virus in mice. J Virol. 2015;90:1872–9. PubMed https://doi.org/10.1128/JVI.02387-15

12. Yamayoshi S, Yamada S, Fukuyama S, Murakami S, Zhao D, Uraki R, et al. Virulence-affecting amino acid changes in the PA protein of H7N9 influenza A viruses. J Virol. 2014;88:3127–34. PubMed https://doi.org/10.1128/JVI.03155-13

13. Hu M, Chu H, Zhang K, Singh K, Li C, Yuan S, et al. Amino acid substitutions V63I or A37S/I61T/V63I/V100A in the PA N-terminal domain increase the virulence of H7N7 influenza A virus. Sci Rep. 2016;6:37800. PubMed https://doi.org/10.1038/srep37800

14. Song J, Feng H, Xu J, Zhao D, Shi J, Li Y, et al. The PA protein directly contributes to the virulence of H5N1 avian influenza viruses in domestic ducks. J Virol. 2011;85:2180–8. PubMed https://doi.org/10.1128/JVI.01975-10

15. Hulse-Post DJ, Franks J, Boyd K, Salomon R, Hoffmann E, Yen HL, et al. Molecular changes in the polymerase genes (PA and PB1) associated with high pathogenicity of H5N1 influenza virus in mallard ducks. J Virol. 2007;81:8515–24. PubMed https://doi.org/10.1128/JVI.00435-07

16. Chen L, Wang C, Luo J, Li M, Liu H, Zhao N, et al. Amino acid substitution K470R in the nucleoprotein increases the virulence of H5N1 influenza A virus in mammals. Front Microbiol. 2017;8:1308. PubMed https://doi.org/10.3389/fmicb.2017.01308

17. Fan S, Deng G, Song J, Tian G, Suo Y, Jiang Y, et al. Two amino acid residues in the matrix protein M1 contribute to the virulence difference of H5N1 avian influenza viruses in mice. Virology. 2009;384:28–32. PubMed https://doi.org/10.1016/j.virol.2008.11.044

18. Jiao P, Tian G, Li Y, Deng G, Jiang Y, Liu C, et al. A single-amino-acid substitution in the NS1 protein changes the pathogenicity of H5N1 avian influenza viruses in mice. J Virol. 2008;82:1146–54. PubMed https://doi.org/10.1128/JVI.01698-07

19. Ayllon J, Domingues P, Rajsbaum R, Miorin L, Schmolke M, Hale BG, et al. A single amino acid substitution in the novel H7N9 influenza A virus NS1 protein increases CPSF30 binding and virulence. J Virol. 2014;88:12146–51. PubMed https://doi.org/10.1128/JVI.01567-14

Appendix Table 1. Surveillance for AIV viruses in Qinghai Lake, 2019–2023

Collection date		Sampling sites	Host	Sample type	Sample size	Influenza A positive	Subtype
2019	5/13	Hada Shoal	Bar-headed goose	Feces	301	8	H2N3
		Egg Island	Bar-headed goose	Feces	257	NA	NA
		Rescue center	Bar-headed goose	Feces	126	NA	NA
		Sheng River estuary	Bar-headed goose	Feces	130	NA	NA
	5/15	Egg Island	Bar-headed goose	Feces	69	NA	NA
	7/1	Egg Island	Bar-headed goose	Feces	39	NA	NA
		Sheng River estuary	Bar-headed goose	Feces	221	NA	NA
	7/2	Hada Shoal	Bar-headed goose	Feces	67	NA	NA
	7/3	Rescue center	Bar-headed goose	Feces	38	NA	NA
	7/4	Black horse river	Bar-headed goose	Feces	135	NA	NA
		Buha River estuary	Bar-headed goose	Feces	279	NA	NA
			Subtotals		1662	8	
2020	7/17	Hada Shoal	Bar-headed goose	Feces	200	NA	NA
	7/18	Buha River estuary	Bar-headed goose	Feces	173	NA	NA
	7/19	Sheng River estuary	Bar-headed goose	Feces	258	NA	NA
	7/2	Black horse River	Bar-headed goose	Feces	45	NA	NA

Collection date		Sampling sites	Host	Sample type	Sample size	Influenza A positive	Subtype
	8/11	Haixin Mountains	Bar-headed goose	Swabs	2	NA	NA
		Haixin Mountains	Bar-headed goose	Feces	68	NA	NA
	8/12	Hada Shoal	Bar-headed goose	Feces	223	NA	NA
	8/13	Buha River estuary	Bar-headed goose	Feces	121	NA	NA
		Sheng River estuary	Bar-headed goose	Feces	157	NA	NA
		Subtotals			1247	0	NA
2021	5/13	Sheng River estuary	Bar-headed goose	Feces	206	NA	NA
	5/14	Hada Shoal	Bar-headed goose	Feces	205	NA	NA
	5/15	Black horse River	Bar-headed goose	Feces	105	NA	NA
	5/16	Egg Island	Bar-headed goose	Feces	146	NA	NA
	6/17	Egg Island	Common Cormorant	Feces	97	NA	NA
	6/18	Hada Shoal	Bar-headed goose	Feces	221	NA	NA
		Buha River estuary	Common Cormorant	Feces	84	NA	NA
	6/19	Hada Shoal	Bar-headed goose	Feces	195	NA	NA
	7/29	Hada Shoal	Bar-headed goose	Feces	29	NA	NA
	7/30	Rescue center	Bar-headed goose	Feces	49	NA	NA
		Sheng River estuary	Bar-headed goose	Feces	133	NA	NA
	7/31	Jiangxi ditch	Bar-headed goose	Feces	80	NA	NA
			Ruddy Shelduck	Feces	350	1	H7N3
		Subtotals			1900	1	
2022	6/18	Sheng River estuary	Bar-headed goose	Feces	160	8	H5N1
			Bar-headed goose	Swabs	3	NA	NA
	6/19	Black horse River	Ruddy Shelduck	Feces	55	NA	NA
		Jiangxi ditch	Ruddy Shelduck	Feces	210	2	H10N7
			Bar-headed goose	Feces	61	1	H10N7
	6/20	Hada Shoal	Brown-headed gull	Swabs	3	1	H2N3
		Hada Shoal	Brown-headed gull	Feces	120	2	H3N8
	6/21	Hada Shoal	Brown-headed gull	Feces	120	3	H10N7
	7/20	Sheng River estuary	Ruddy Shelduck	Feces	211	NA	NA
	7/22	Hada Shoal	Bar-headed goose	Feces	61	NA	NA
		Hada Shoal	Ruddy Shelduck	Feces	69	2	H5N1
	7/23	Black horse River	Brown-headed gull	Feces	48	NA	NA
			Bar-headed goose	Feces	30	NA	NA
		Jiangxi ditch	Ruddy Shelduck	Feces	210	NA	NA
			Bar-headed goose	Feces	33	3	H5N1
	7/24	Egg Island	Brown-headed gull	Feces	68	NA	NA
		Subtotals			1462	22	NA

Collection date		Sampling sites	Host	Sample type	Sample size	Influenza A positive	Subtype
2023	5/14	Hada Shoal	Ruddy Shelduck	Feces	375	NA	NA
	5/15	Hada Shoal	Ruddy Shelduck	Feces	75	NA	NA
			Bar-headed goose	Feces	8	NA	NA
			Snipe	Feces	99	NA	NA
	5/16	Hada Shoal	Bar-headed goose	Feces	255	NA	NA
	5/16	Sheng River estuary	Bar-headed goose	Feces	86	NA	NA
	5/18	Egg Island	Bar-headed goose	Feces	73	NA	NA
			Common Cormorant	Feces	13	NA	NA
	6/15	Hada Shoal	Bar-headed goose	Feces	290	NA	NA
	6/16	Hada Shoal	Ruddy Shelduck	Feces	345	NA	NA
		Sheng River estuary	Bar-headed goose	Feces	290	NA	NA
	6/17	Na Ren Wetland	Bar-headed goose	Feces	350	NA	NA
	7/19	Hada Shoal	Bar-headed goose	Feces	200	NA	NA
		Sheng River estuary	Bar-headed goose	Feces	212	NA	NA
	7/20	Sheng River estuary	Bar-headed goose	Feces	162	NA	NA
		Na Ren Wetland	Bar-headed goose	Feces	73	NA	NA
	7/21	Jiangxi ditch	Ruddy Shelduck	Feces	394	3	H4N6
						4	H10N1
						3	H10N5
						5	H10N9
						2	H12N5
		Black horse River	Bar-headed goose	Feces	90	NA	NA
	9/17	Hada Shoal	Ruddy Shelduck	Feces	37	NA	NA
		Sheng River estuary	Bar-headed goose	Feces	36	NA	NA
	9/18	Jiangxi ditch	Ruddy Shelduck	Feces	18	NA	NA
		Subtotals			3481	17	NA
Total					9752	48	NA

Appendix Table 2. Detailed information of tissue samples collected from wild birds in Qinghai Lake

Number	Host	Sites	Time	Tissue sampled	Influenza A positive
1	Brown-headed gull	Hada Shoal	2022/7/21	Heart, liver, lung, intestine, brain	H5N1
2	Brown-headed gull	Hada Shoal	2022/7/21	Heart, liver, lung, kidney, intestine, brain	H5N1
3	Crested grebe	Egg Island	2022/7/21	Heart, liver, lung, kidney, intestine, brain	H5N1
4	Crested grebe	Egg Island	2022/7/21	Heart, lung	H5N1
5	Brown-headed gull	Hada Shoal	2022/7/22	Heart, liver, lung, kidney, intestine, brain	H5N1
6	Brown-headed gull	Hada Shoal	2022/7/22	Heart, liver, lung, kidney, intestine, brain	H5N1
7	Brown-headed gull	Sheng River estuary	2022/7/20	Heart, liver, lung, intestine, brain	H5N1
8	Crested grebe	Ga ri la	2022/7/15	Heart, liver, lung, kidney, intestine, brain	H5N1
9	Crested grebe	Ga ri la	2022/7/15	Heart, liver, lung, brain	H5N1
10	Crested grebe	Ga ri la	2022/7/15	Heart, liver, lung, intestine, brain	H5N1
11	Brown-headed gull	Black horse River	2022/7/23	Heart, liver, lung, intestine, brain	H5N1
12	Brown-headed gull	Black horse River	2022/7/23	Heart, liver, lung, brain	H5N1
13	Brown-headed gull	Hada Shoal	2023/5/15	Heart, liver, lung, kidney, spleen, brain	NA
14	Bar-headed goose	Sheng River estuary	2023/5/16	Heart, liver, lung, kidney, intestine, brain, spleen	NA
15	Yellow-bellied Tit	Sheng River estuary	2023/5/16	Heart, liver, lung, intestine, brain	NA
16	Cormorant	Egg Island	2023/5/18	Heart, liver, lung, intestine, brain	NA
17	Cormorant	Egg Island	2023/5/18	Heart, liver, lung, intestine, kidney, brain	NA
18	Cormorant	Egg Island	2023/5/18	Heart, liver, lung, intestine, kidney, brain	NA
19	Sparrow	Hada Shoal	2023/6/16	Heart, liver, lung, intestine, kidney, brain	NA
20	Wild duck	Hada Shoal	2023/6/16	Heart, liver, lung, intestine, kidney, brain	NA
21	Bar-headed goose	Sheng River estuary	2023/7/19	Heart, liver, lung, intestine, kidney, brain	NA

Appendix Table 3. Amino acid residues of Qinghai H5N1 viruses associated with increased human-type receptor binding or promoting the replication, virulence, and transmission of avian influenza viruses in mammalian hosts.

Protein	Amino acid	G1†	G2†	Phenotypic consequences
HA*	Cleavage site	PLREKRRKR/G	PLREKRRKR/G	Feature of HPAIV (*5*)
	T160A	A	A	Increases virus binding to the human receptor (α-2, 6) (*6*, *7*)
PB2	I495V	V	V	Increases virulence and replication in mammals (*8*).
	A676T	T	T	
	L89V	V	V	Increases polymerase activity in mammals (*8*, *9*)
	G309D	D	D	
	R477G	G	G	
	T339K	K	K	
	A655V	V	V	
PB1	D3V	V	V	Enhances viral polymerase activity (*10*)
	D622G	G	G	Enhances viral polymerase activity, virulence in mice (*11*)
PA	S37A	A	A	Increases polymerase activity in human cells (*12*)
	V63I	I	V	Increases viral polymerase activity, viral replication and virulence in mammalian cells (*13*)
	N383D	D	D	Increases polymerase activity in human cells (*14*, *15*)
	S515T	T	T	
NP	K470R	R	R	Increases virus infectivity and virulence increase (*16*)
M	N30D	D	D	Increases viral replication and viral virulence in mice (*17*)
	T215A	A	A	
NS1	P42S	S	S	Increases virulence and decreased antiviral response in mice (*18*)
	I106M	M	M	Increases viral virulence in mice by enhanced CPSF30 binding (*19*)

*The numbering of HA is relative to A/New York/392/2004(H3N2).

†Genotype 1 and 2 of Qinghai H5N1 viruses isolated in 2022.

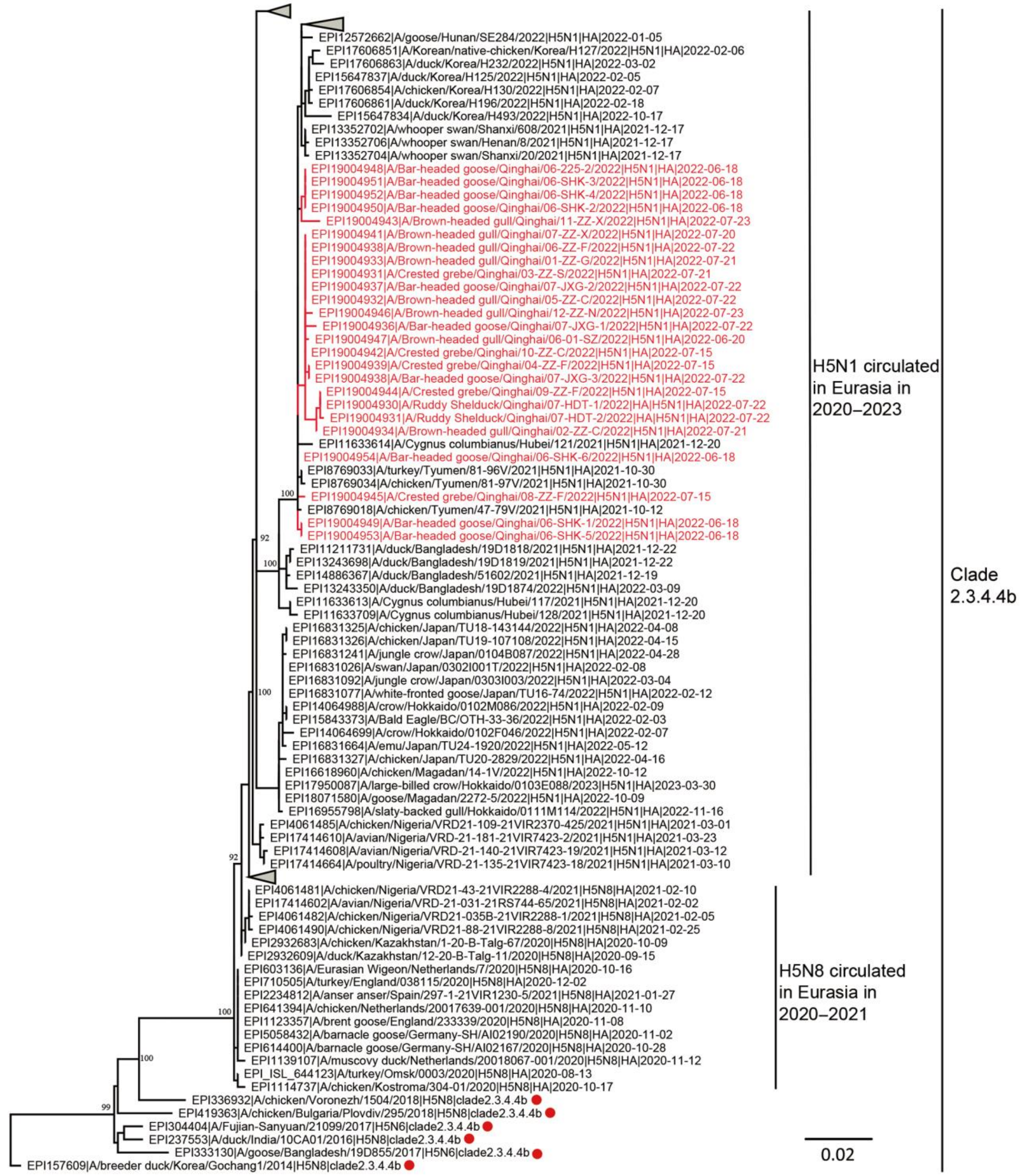

Appendix Figure 1. Maximum-likelihood phylogenetic trees for hemagglutinin gene segments of Qinghai Lake H5N1, China, 2022. Blue circle, the branch of clade 2.3.4, clade 2.3.4.4a, and c-h; red spot, the branch of clade 2.3.4.4b viruses; red text, virus strain names of Qinghai Lake H5N1. The scale bar represents nucleotide substitutions per site.

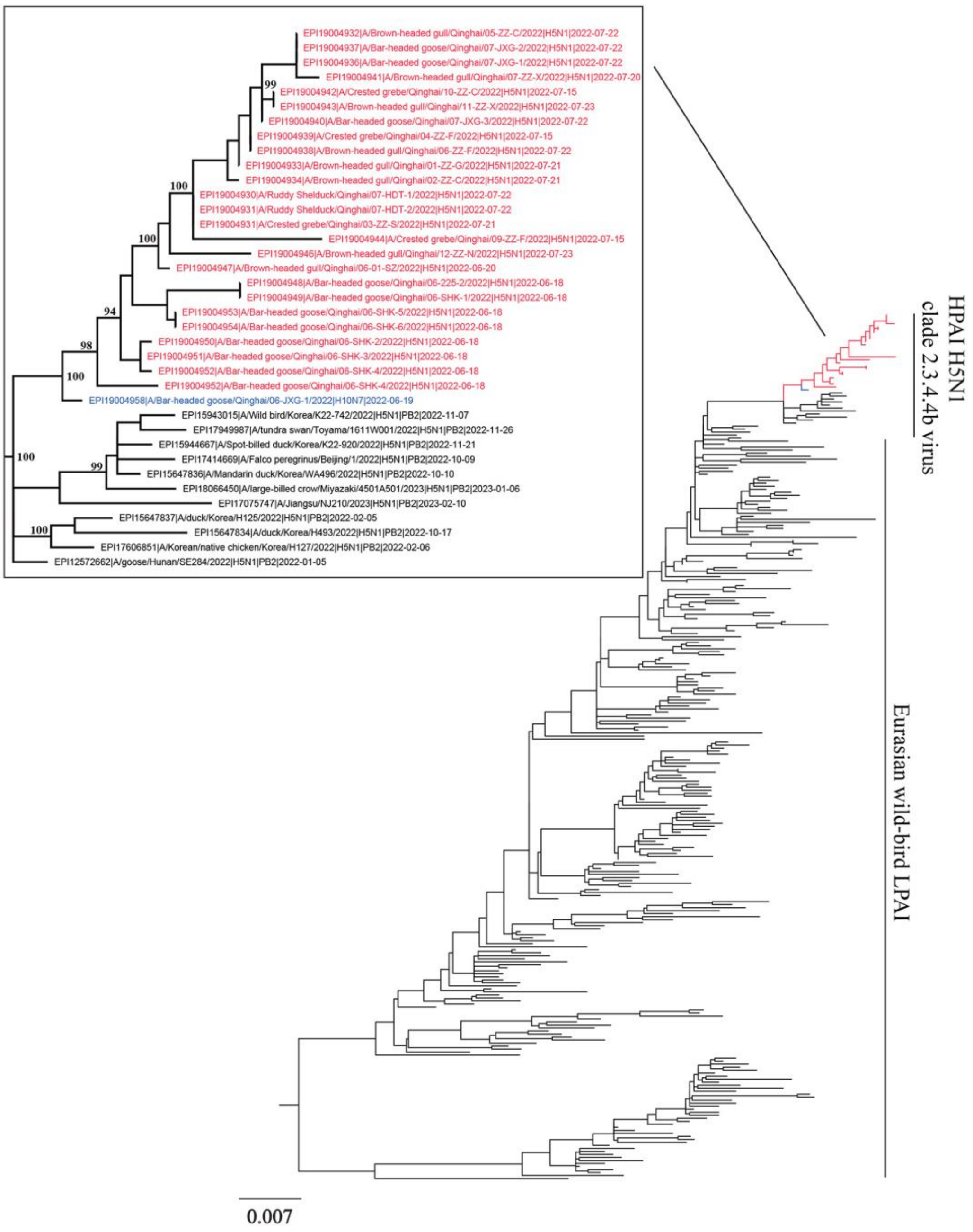

Appendix Figure 2. Maximum-likelihood phylogenetic trees of the coding sequences of polymerase basic protein 2 segments. Node labels indicate bootstrap values. Red, Qinghai Lake H5N1 strains; blue, Qinghai Lake H10N7 strain. The scale bar represents nucleotide substitutions per site.

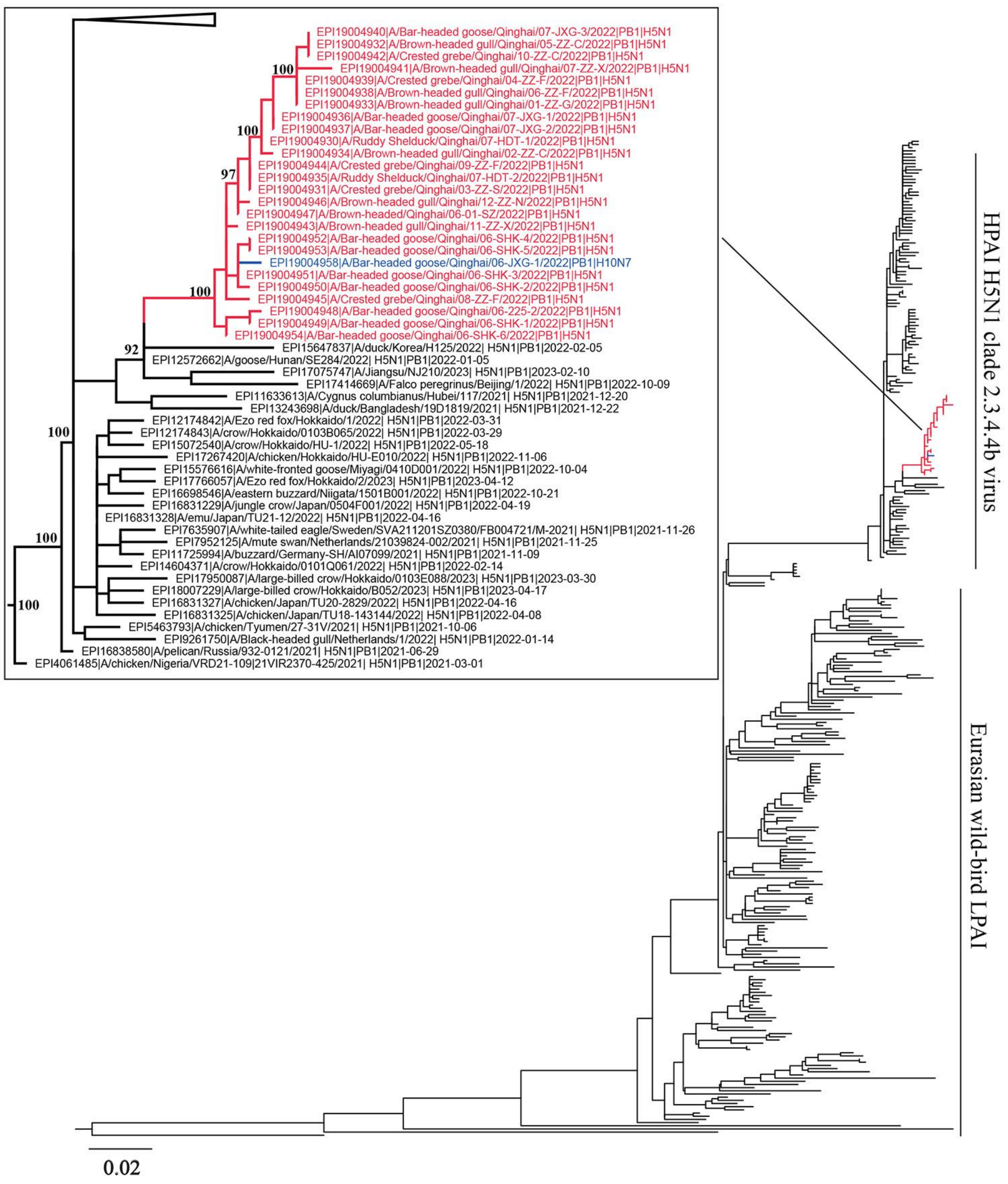

Appendix Figure 3. Maximum-likelihood phylogenetic trees of the coding sequences of polymerase basic protein 1 segments. Node labels indicate bootstrap values. Red, Qinghai Lake H5N1 strains; blue, Qinghai Lake H10N7 strain. The scale bar represents nucleotide substitutions per site.

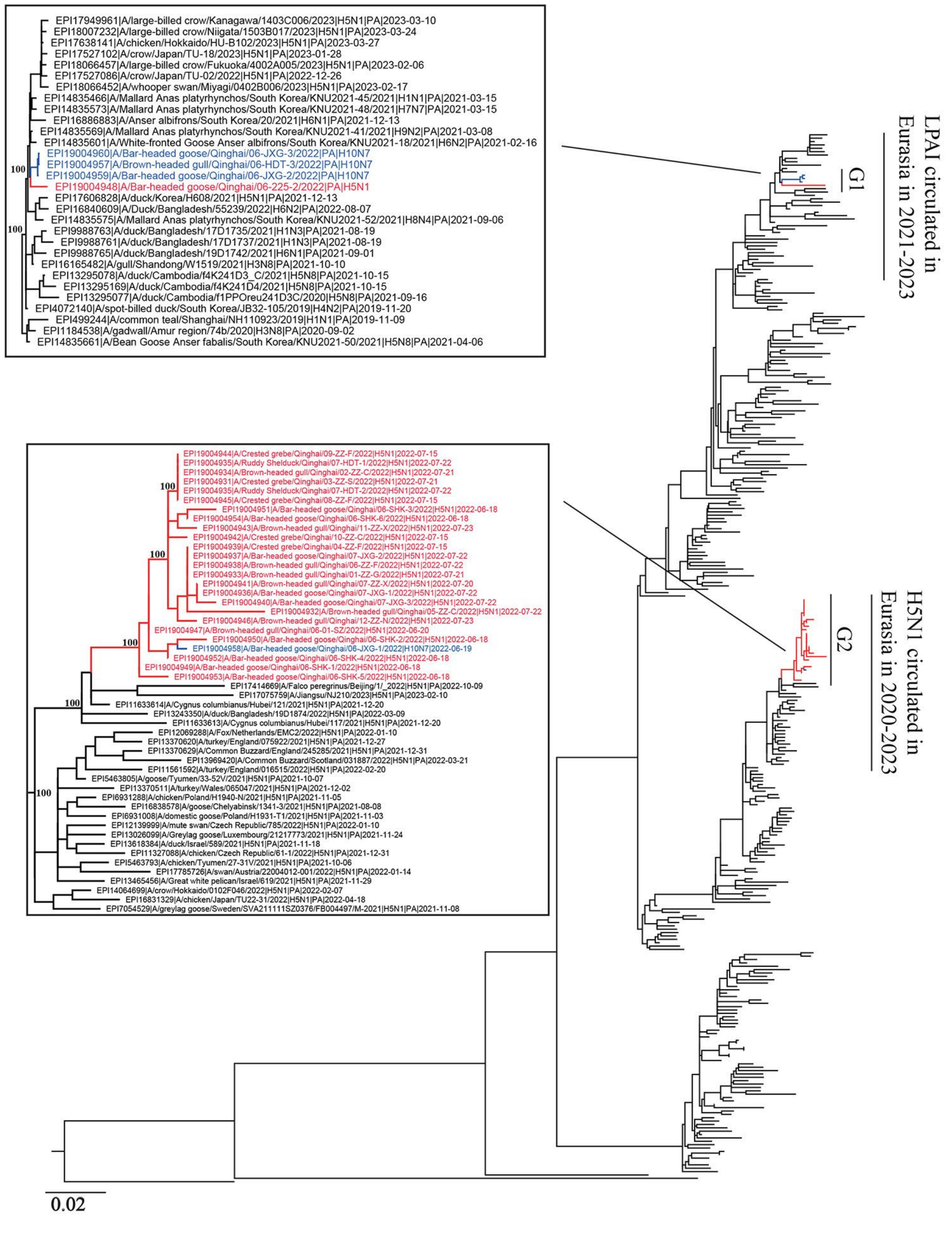

Appendix Figure 4. Maximum-likelihood phylogenetic trees of the coding sequences of polymerase acidic protein segments. Node labels indicate bootstrap values. Red, Qinghai Lake H5N1 strains; blue, Qinghai Lake H10N7 strain. The scale bar represents nucleotide substitutions per site.

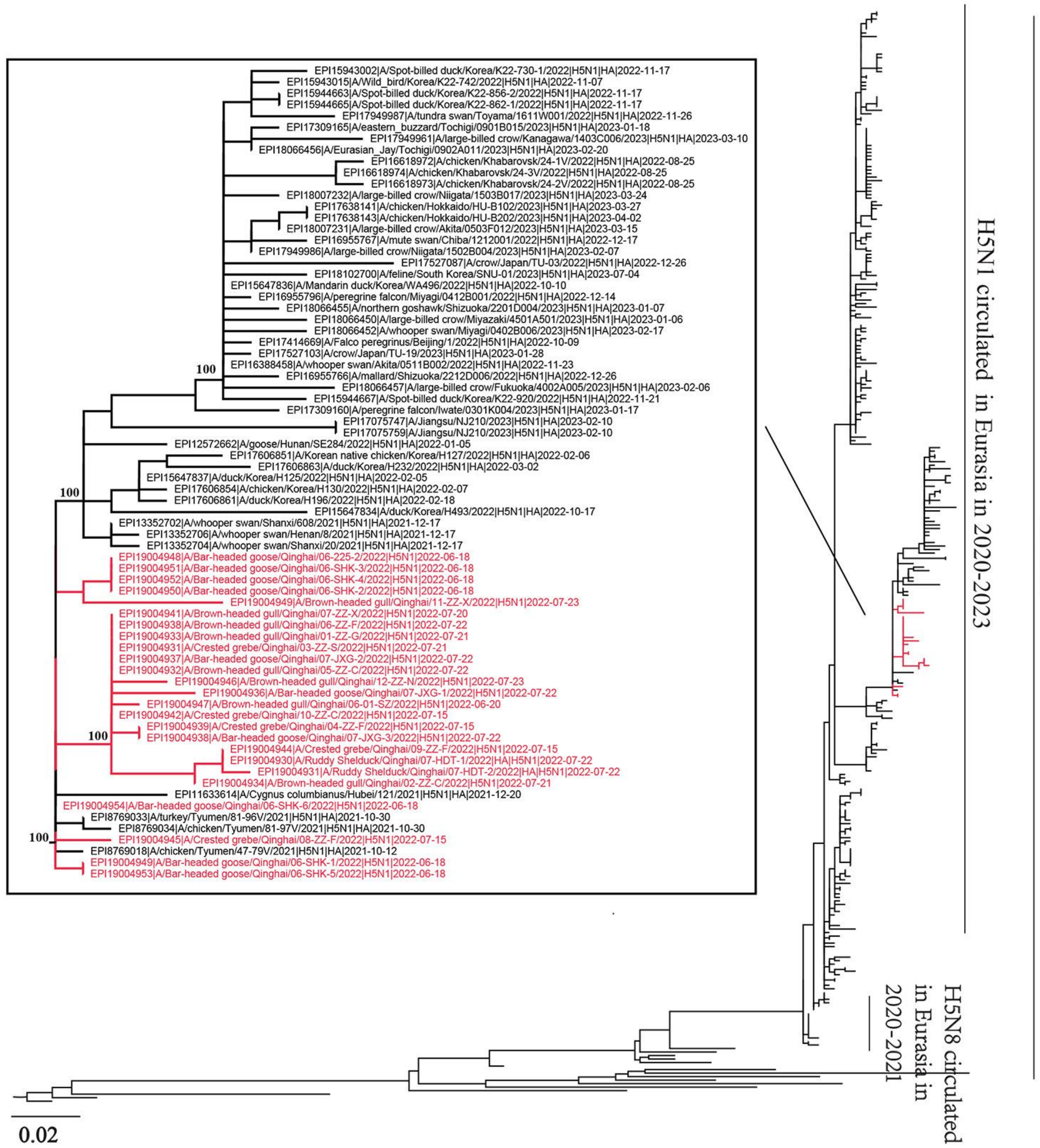

Appendix Figure 5. Maximum-likelihood phylogenetic trees of the coding sequences of hemagglutinin segments. Node labels indicate bootstrap values. Red, Qinghai Lake H5N1 strains; blue, Qinghai Lake H10N7 strain. The scale bar represents nucleotide substitutions per site.

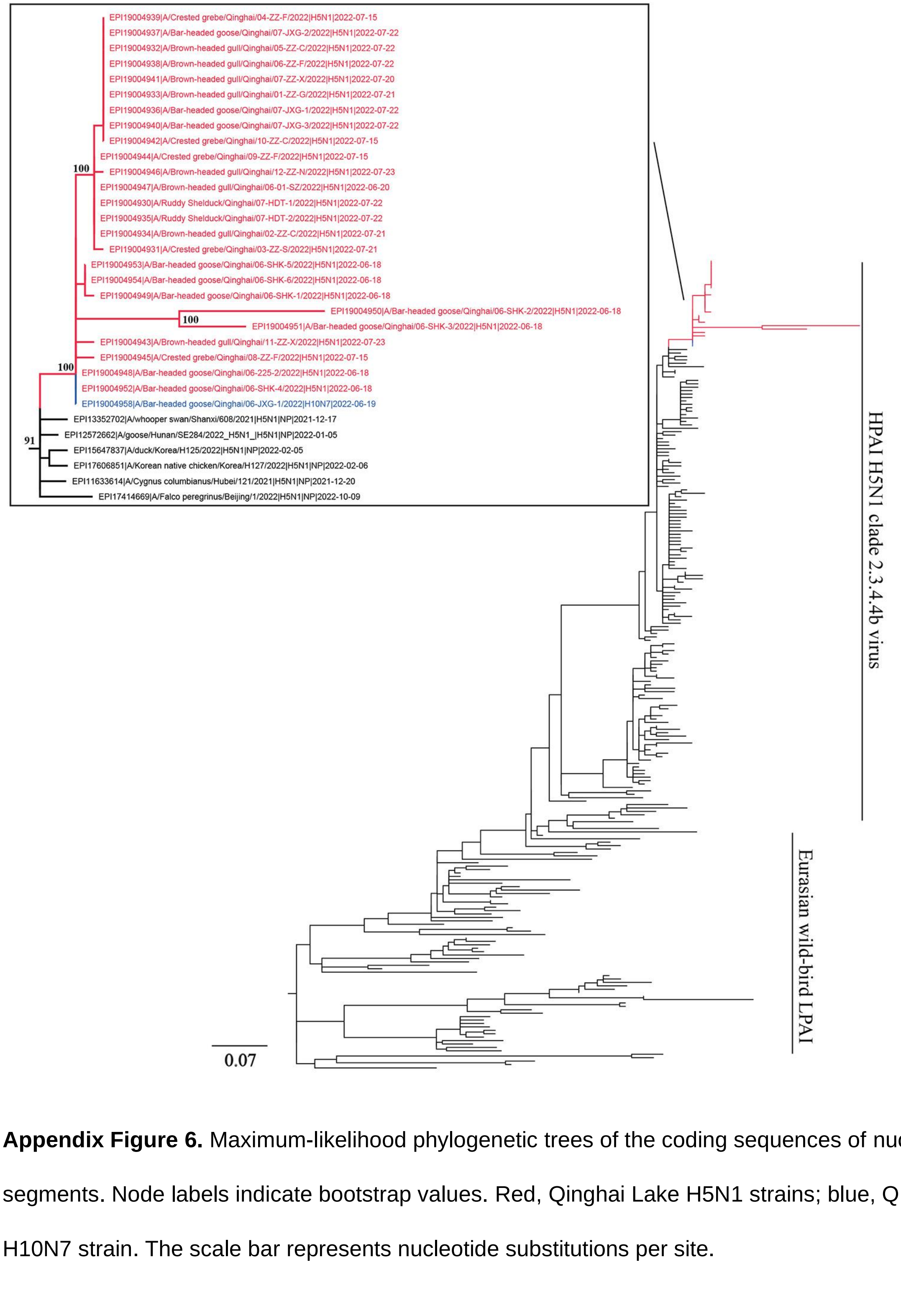

Appendix Figure 6. Maximum-likelihood phylogenetic trees of the coding sequences of nucleoprotein segments. Node labels indicate bootstrap values. Red, Qinghai Lake H5N1 strains; blue, Qinghai Lake H10N7 strain. The scale bar represents nucleotide substitutions per site.

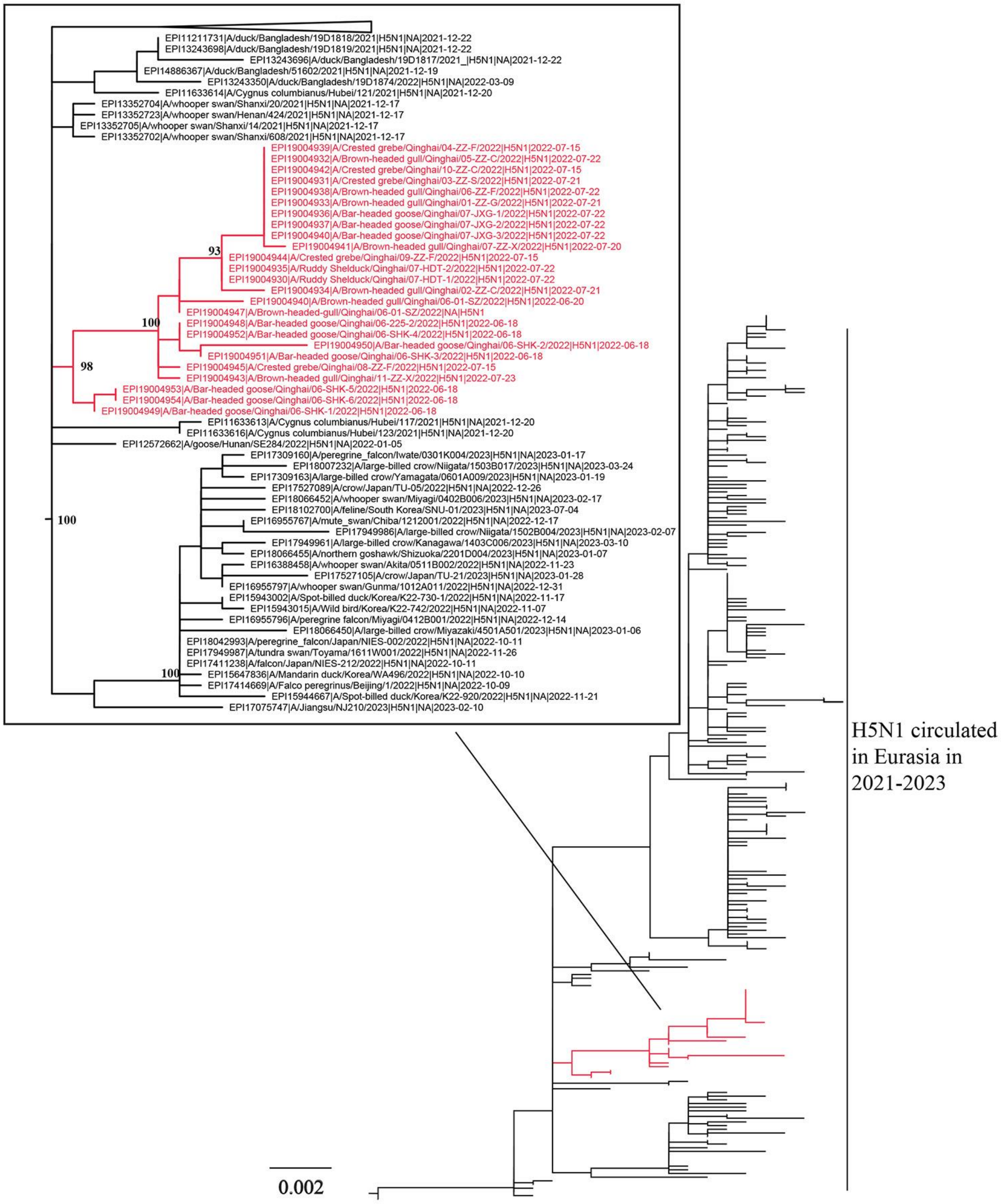

Appendix Figure 7. Maximum-likelihood phylogenetic trees of the coding sequences of neuraminidase segments. Node labels indicate bootstrap values. Red, Qinghai Lake H5N1 strains; blue, Qinghai Lake H10N7 strain. The scale bar represents nucleotide substitutions per site.

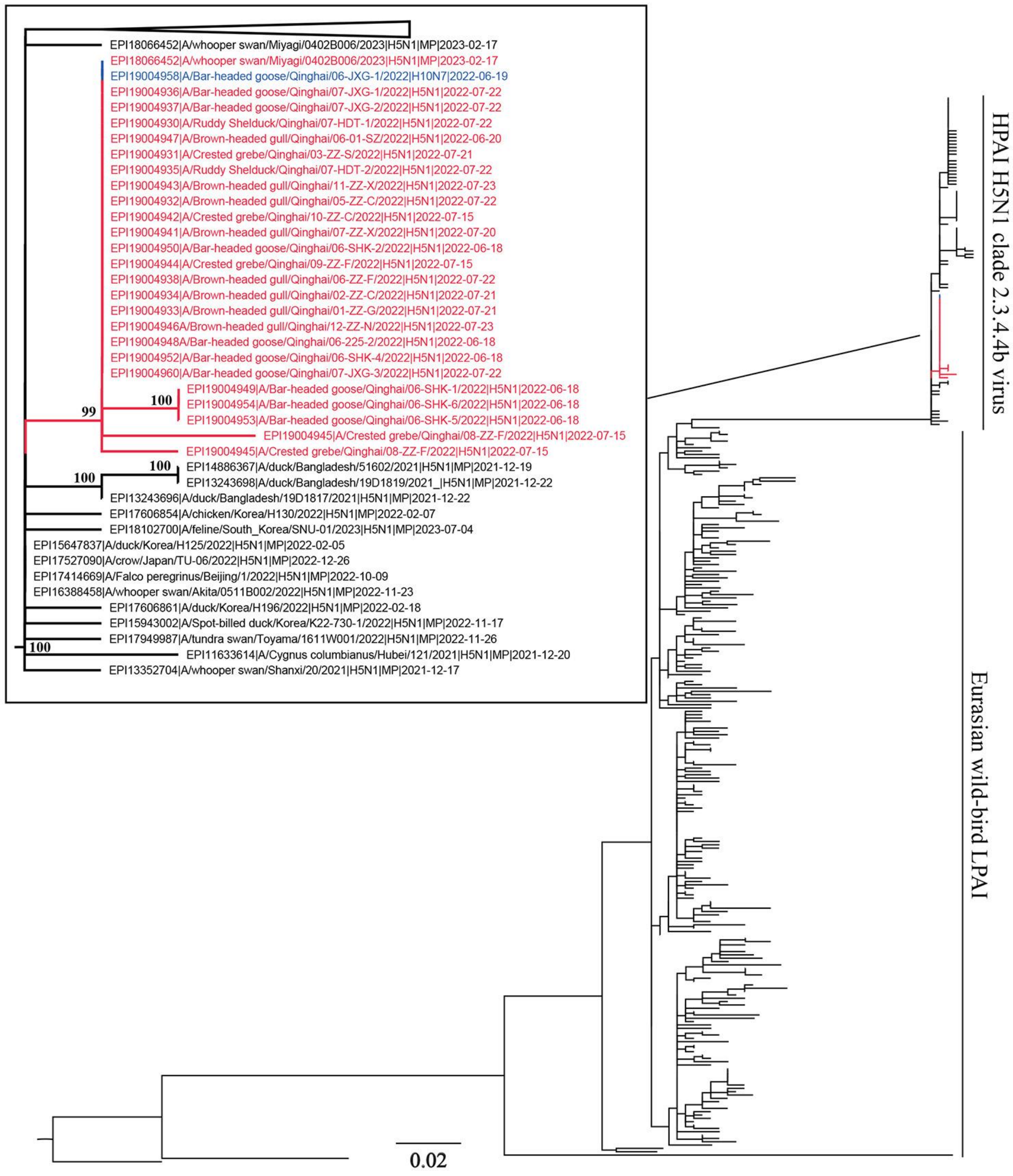

Appendix Figure 8. Maximum-likelihood phylogenetic trees of the coding sequences of matrix protein segments. Node labels indicate bootstrap values. Red, Qinghai Lake H5N1 strains; blue, Qinghai Lake H10N7 strain. The scale bar represents nucleotide substitutions per site.

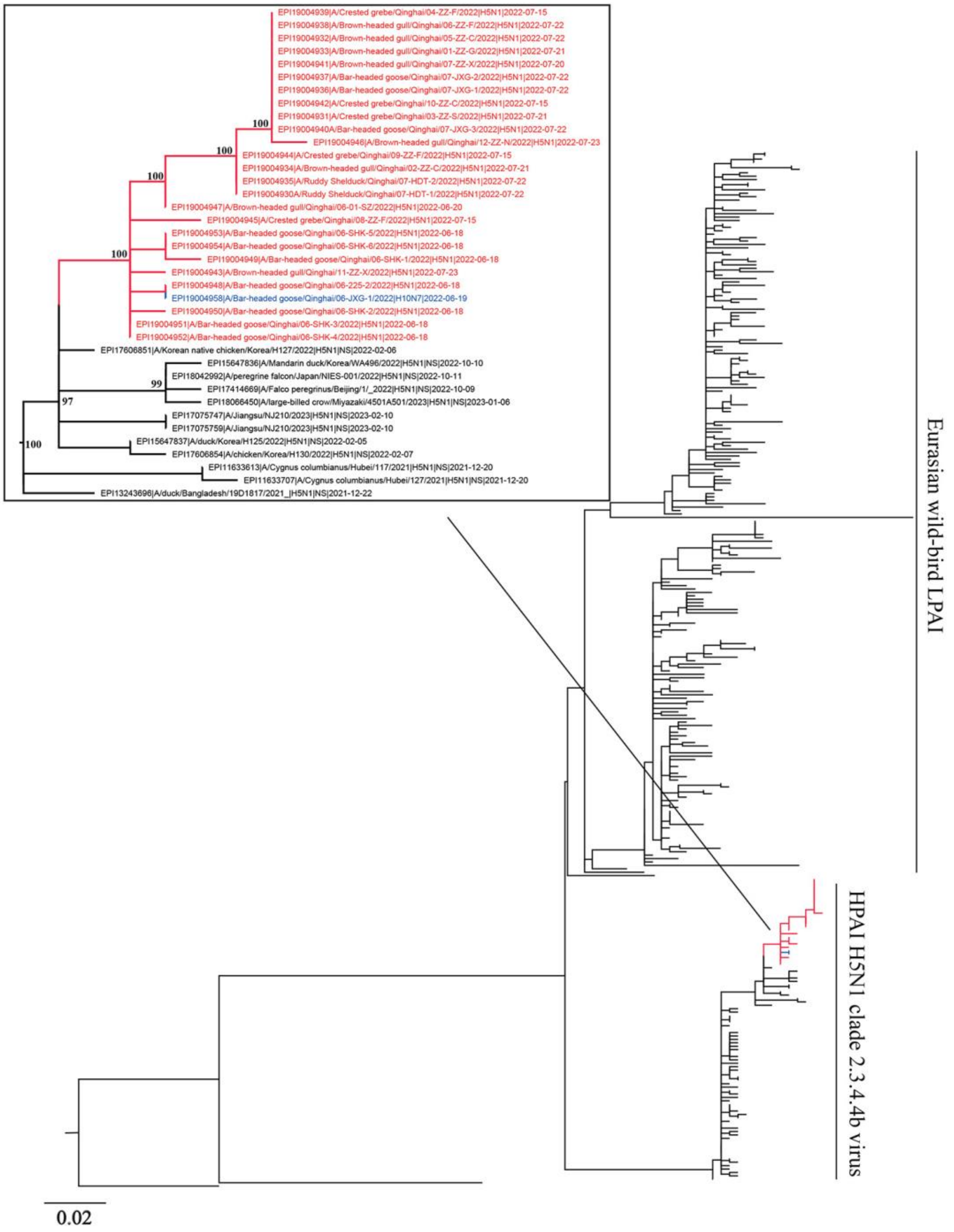

Appendix Figure 9. Maximum-likelihood phylogenetic trees of the coding sequences of nonstructural protein segments. Node labels indicate bootstrap values. Red, Qinghai Lake H5N1 strains; blue, Qinghai Lake H10N7 strain. The scale bar represents nucleotide substitutions per site.
